# Manipulation of cell membrane using carbon nanotube scaffold as a photoresponsive stimuli generator

**DOI:** 10.1088/1468-6996/15/4/045002

**Published:** 2014-07-07

**Authors:** Takao Sada, Tsuyohiko Fujigaya, Naotoshi Nakashima

**Affiliations:** 1 Department of Applied Chemistry, Graduate School of Engineering, Kyushu University, 744 Motooka Nishi-ku, Fukuoka 819-0395, Japan; 2 International Institute for Carbon-Neutral Energy Research (WPI-I2CNER), Kyushu University, Japan; 3 Core Research for Evolutionary Science and Technology (JST-CREST), 5 Sanbancho, Chiyoda-ku, Tokyo, 102-0075, Japan

**Keywords:** carbon nanotubes, cell scaffold, cell perforation, nanosecond pulse laser, near-IR laser

## Abstract

We describe, for the first time, the perforation of the cell membrane in the targeted single cell based on the nanosecond pulsed near-infrared (NIR) laser irradiation of a thin film of carbon nanotubes that act as an effective photon absorber as well as stimuli generator. When the power of NIR laser is over 17.5 *μ*J/pulse, the cell membrane after irradiation is irreversibly disrupted and results in cell death. In sharp contrast, the perforation of the cell membrane occurs at suitable laser power (∼15 *μ*J/pulse) without involving cell termination.

## 1. Introduction

In current cell engineering and tissue biology research, pulsed laser-mediated stimulation of a cell has emerged as a powerful technique for various targets, such as selective gene transfection [1], drug injection [2], regulation of gene expression [3, 4], etc, due to its high selectivity together with controllability based on the nature of the laser. Irradiation of biological cells by a pulsed laser induces the perforation (ablation) of the cell membrane based on the generated stimuli, such as low-density plasma, cavitation nano-bubbles and shock-wave [1]. Such damage of the membrane integrity significantly accelerates the transfection of genes and the delivery of drugs into the cells. The high selectivity of the laser-based perforation and successive internalization of the foreign molecules (optoporation) [5–7] provides a sharp contrast to other approaches, such as electroporation [8–10], the gene gun [11], chemical transfection [12] and virus-mediated transduction [13], all of which enable the transformation of a large population of cells in an untargeted manner.

Among the wide range of laser energies, the near-infrared (NIR) region has been proven to be much less harmful for biological cells because of their almost non-absorption in the NIR wavelength region [14]. In particular, the most successful NIR lasers have been reported to be femtosecond lasers due to their fine spatial resolution of the ablation area with no thermal or mechanical damage to surrounding materials together with a low energy threshold for the ablation [15].

However, femtosecond laser instruments are expensive and require a highly sophisticated optical arrangement and large space. For these reasons, in fundamental cell and tissue biology research and also in their applications, a relatively inexpensive and easy-to-use technique that can be easily integrated into an existing microscope system is strongly required. Recently, to increase the accessibility of the laser-mediated stimulation and decrease the cost, the use of a nanosecond laser was proposed due to its compactness and lower cost than that of a femtosecond system [16, 17].

One of the main issues when switching from a femtosecond laser to nanosecond laser is the linear dependency of the absorption coefficients of the target materials on the excitation [18], which suggested that strong light absorption is required for effective ablation. However, the number of organic molecules having a strong absorbance in the NIR region is very limited. In addition, without a strong absorption, a much higher energy is required of nanosecond duration to achieve a high intensity of laser flux compared to the femtosecond duration. Therefore, an extremely strong light absorbance is necessary to minimize the laser intensity for the stimulation induced by the nanosecond pulse laser.

To satisfy such a requirement, the use of gold nanoparticles (Au-NP) and carbon nanotubes (CNTs) as a photon absorbing material has recently attracted considerable attention due to their characteristic strong absorption coefficients in the NIR region; in fact, they have been used as effective photon-to-stimulus converters [19, 20]. Lalonde *et al* reported an NIR nanosecond laser-mediated optoporation using Au-NP [17], where Au-NP dispersed in the cultured media absorbed NIR laser due to the strong absorption and induced the perforation by generated stimuli such as cavitation nano-bubbles and heat. We reported a selective single cell catapulting through the NIR irradiation using a cell culture dish coated with single-walled carbon nanotubes (SWCNTs). An effective absorption of the NIR light for the SWCNTs allowed us to use a nanosecond NIR laser [21], in which an effective shock-wave generation from the irradiated SWCNTs played a key role in catapulting the targeted cell [22].

CNTs have received a great deal of interest in the biomedical fields in recent years [23–25] especially as materials for tissue engineering scaffolds [26, 27]. The use of CNTs as cell scaffold materials has been shown to support the adhesion, growth, differentiation and spheroid configuration of cultured cells because of their structural similarity to the fibrous network structures formed by the CNTs with the natural extracellular matrices [28–31]. Furthermore, their high electrical conductivity enables us to monitor the dynamic secretion from the living cells in real time [32], to stimulate the cells by the electrical signal [33] and to improve the neuronal recordings [34].

In this study, we describe a potential use of an SWCNT-coated dish as an antenna for the NIR nanosecond pulse laser for perforation of the cell membranes. Compared to Au-NP that was dispersed in medium solutions, the SWCNTs are tightly immobilized on the substrate in the SWCNT-coated dish, and the contamination of the cell due to the internalization of the antenna materials is avoidable. In addition, an effective perforation with an ultralow energy is expected to be induced due to the strong absorption of the SWCNT in the NIR region.

## 2. Experimental details

### 2.1. Materials

SWCNTs (synthesized by an arc discharge method) were purchased from Meijo Nano Carbon. Carboxymethyl cellulose sodium salt (CMC-Na) was purchased from Kishida Chemical and used as received.

### 2.2. Fabrication of an SWCNT-coated dish

CMC-Na (3 mg) and SWCNTs (1 mg) were dissolved in H_2_O (10 mL) by sonication using a bath-type ultrasonicator (Branson 5510) for 120 min, followed by centrifugation (10 000 g) using a high-speed centrifuge (Kubota, 3K30C) for 15 min. The obtained SWCNT aqueous dispersion was sprayed at 100 °C onto a glass substrate (Glass Base Dish, IWAKI, 27 mm *φ*) placed on a temperature-controlled sheet. The amount of the SWCNTs on the glass dish was controlled by the repeated spraying numbers. Each spraying cycle was carried out by spraying the SWCNTs dispersion onto a glass-bottom dish for ∼1 s, then drying ∼9 s. The obtained SWCNT-coated dish was then immersed in deionized water and dried under flowing N_2_ gas. The dish was sterilized by ultraviolet radiation for 24 h prior to the cell culture. The absorption spectra of the SWCNT-coated dish were recorded using a UV/Vis/NIR spectrophotometer (JASCO, V-570).

### 2.3. Cell culture

HeLa cells were seeded on the SWCNT-coated dish. The cells were cultured in D-MEM (Wako) with a 10% FBS and antibiotic–antimycotic (GIBCO) at 37 °C in humidified 5% CO_2_.

### 2.4. Determination of laser power upon irradiation

The cultured dish was placed on the stage of an inverted microscope (Nikon, TE2000) equipped with a stage-top incubator (Tokai Hit, ONICS). An NIR pulsed laser (Nd:YVO_4_, 1064 nm, 20 Hz, New Wave Research, Polaris III) with a pulse duration of 4 ns was focused on the SWCNT-coated dish through the objective lens (10×: numerical aperture NA = 0.3, 20×: NA = 0.45, 40×: NA = 0.6, 60×: NA = 0.7, 100×: NA = 0.5–1.3) and irradiated with 1 pulse to a single HeLa cell target. The laser power was monitored by an energy meter display (Ophir Optronics, Orion TH) equipped with a thermal head (Ophir Optronics, 30A-P) near each focal point. The microscopy images were monitored by a CCD video camera (Watec, model WAT-221 s). In order to estimate a minimum energy, more than five cells were irradiated at each given laser power.

### 2.5. Cell staining and monitoring the fluorescence

The staining solution (Dojindo, Cellstain Double Staining Kit) prepared by a calcein-acetoxymethyl (calcein-AM) stock solution (10 *μ*L, 1 mmol mL^−1^) and propidium iodide (PI) solution (15 *μ*L, 1.5 mmol mL^−1^) was added to the culture medium (5 mL). The solution was added to the dish and incubated at 37 °C in humidified 5% CO_2_ for 10 min. After staining, the NIR laser was focused on the SWCNT-coated dish through the objective lens (10×: NA = 0.3) and irradiated with 1 pulse to a single HeLa cell. The fluorescence of the irradiated HeLa cell was recorded by a multichannel spectrophotometer for 30 s (Otsuka Electronics, MCPD-7700) (see figure 3(a) in the main body). The fluorescence images were recorded 10 min after the irradiation using the fluorescence filters of B-2A (*λ*_ex_ = 450–490 nm, *λ*_em_ = > 520 nm, Nikon) and G-2A (*λ*_ex_ = 510–560 nm, *λ*_em_ = > 590 nm, Nikon) for the calcein-AM and PI, respectively. Staining with 3,3′-dihexyloxacarbocyanine iodide (DiOC_6_, Enzo Life Science) was conducted by adding the 2 mL DiOC_6_ (5 mM, in dimetyl sulfoxide) to the culture medium (5 mL) followed by incubation at 37 °C in humidified 5% CO_2_ for 20 min.

## 3. Results and discussion

**Figure 1.**
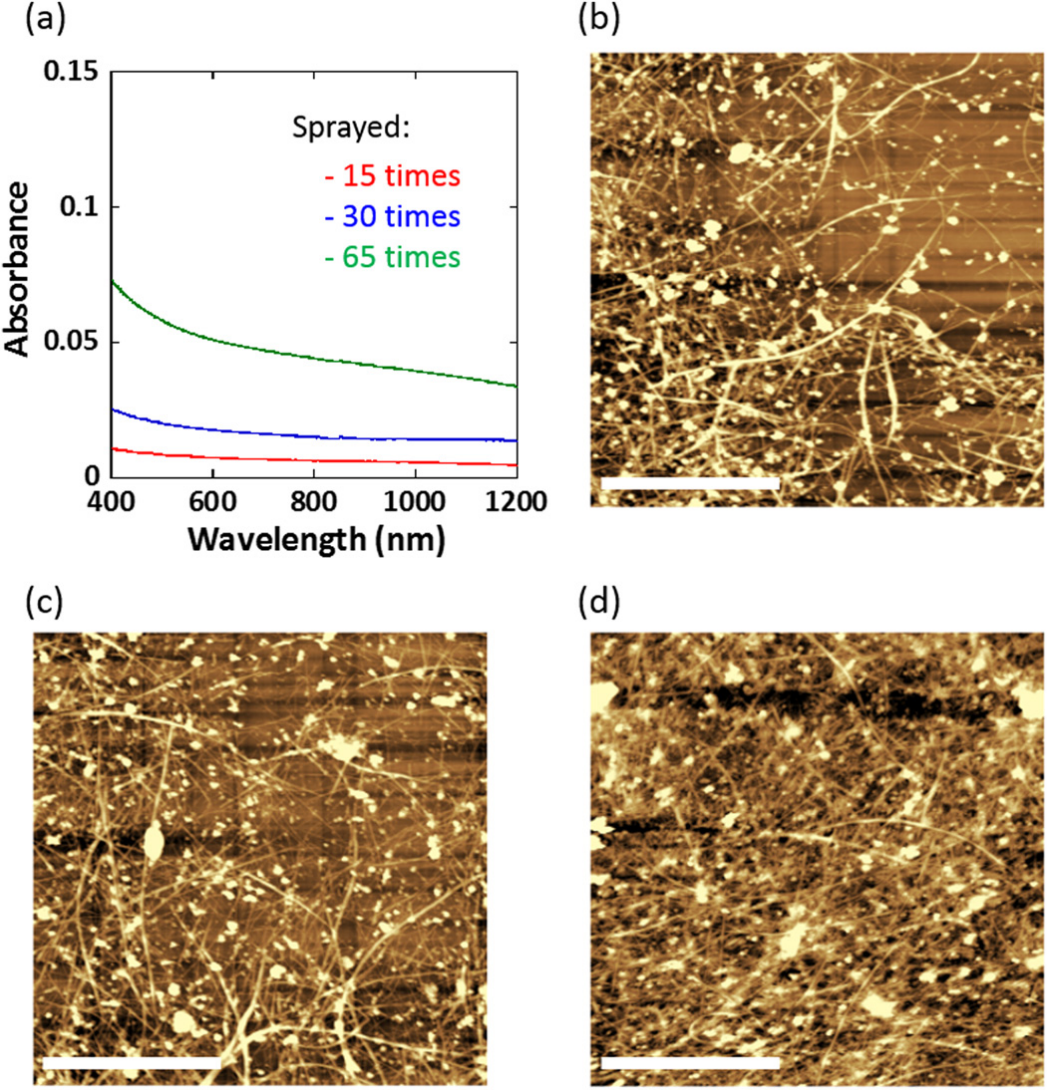
(a) Absorption spectra of the SWCNT-coated dish prepared by spraying 15 (red), 30 (blue) and 65 (green) times. (b)–(d) AFM images of the SWCNT-coated dishes prepared by spraying (b) 15, (c) 30 and (d) 65 times. Scale bar: 2 *μ*m.

The coating of the SWCNTs on a glass-bottom dish was carried out by a spray-coating method according to our previous report [21]. One of the advantages of this method is the good controllability of the density of the SWCNTs on the dish by controlling the number of spraying passes and/or the concentrations of the SWCNT dispersions. We prepared three different SWCNT-coated dishes by changing the number of spray passes. Figure 1(a) shows the absorption spectra of the three SWCNT-coated dishes prepared by spraying 15 (red line), 30 (blue line) and 65 (green line) times, in which the absorbance increased linearly with increasing the number of spraying passes (figure S1, available at stacks.iop.org/STAM/15/045002/mmedia).

The substrate prepared by the spraying 15, 30 and 65 times exhibited a surface resistivity of 1 × 10^5^, 1 × 10^4^ and 3 × 10^3^ *Ω* sq^−1^, respectively. Although the change in the color of the coated dish was visually very subtle (figure S2, available at stacks.iop.org/STAM/15/045002/mmedia), the surface resistivity decreased by two orders of magnitude with increasing number of spraying passes due to the high conductivity of the SWCNTs. Such good controllability, while retaining an excellent transparency ranging from the visible to NIR region as shown in figure 1(a), is crucial for the cell manipulation during the microscopy measurements. The monotonic reduction in optical absorption with increasing wavelength is due to the decrease in the absorption of *π* plasmonic transition of metallic SWCNTs [35]. The morphological image observation of the SWCNT-coated dishes were carried out by atomic force microscopy, and found the formation of fibrous network structures as shown in figures 1(b)–(d). In addition, the surface roughness of the coating layer became higher as the surface resistivity decreased, which suggested that the thickness of the film became thicker with increasing number of spraying passes (for their height profiles, see figure S3, available at stacks.iop.org/STAM/15/045002/mmedia). It is well known that such fibrous network structures are suitable for cell culture scaffolds because of their similarity to a natural extracellular matrix [36] with a fibrous network structure formed by collagen and laminins.

**Figure 2.**
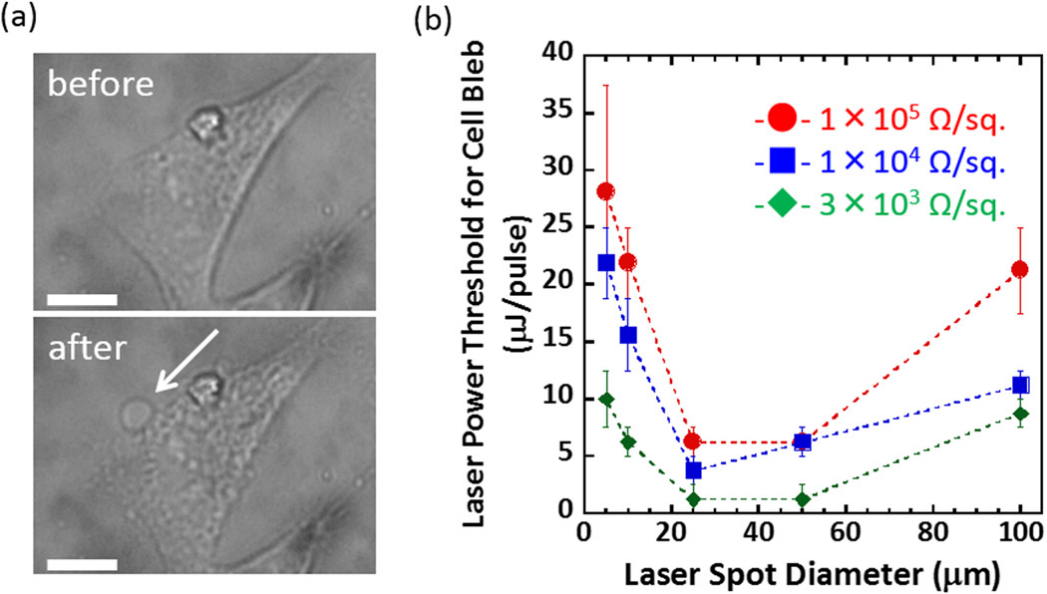
(a) Optical microscopy images of the cells cultured on the SWCNT-coated dish with a resistivity of 1 × 10^4^ *Ω* sq^−1^ before (upper) and after (lower) the irradiation using an objective lens of 60×. The cell after the irradiation shows membrane blebbing as indicated by the white arrow. Scale bar: 10 *μ*m. (b) Plots of the minimum laser energy leading to blebbing depending on laser spot diameter for SWCNT thin films of 1 × 10^5^ (red), 1 × 10^4^ (blue) and 3 × 10^3^ (green) *Ω* sq^−1^ on each dish.

In this study, the HeLa cells were chosen due to their good adhesion onto the substrate as well as their easy cell culture. The cultured cells of interest on the SWCNT-coated dishes were irradiated by the pulsed NIR laser (1 pulse, 1064 nm) through objective lenses of 10×, 20×, 40×, 60× and 100×, corresponding to laser spot diameters of 100, 50, 25, 10 and 5 *μ*m, respectively [21]. We found that the irradiation of the NIR pulse laser often led to bleb growth in the irradiated cell membrane (figure 2(a)) even at a very low energy level (<25 *μ*J/pulse). Blebs are typically observed in two different cases; namely (i) the cell physiology such as the cytokinesis, cell spreading and locomotion or (ii) in the case of cell death such as apoptosis and necrosis [37]. The origin of blebbing is usually attributed to their size, mobility and transparency [37]. In our study, the blebs were relatively large and stably settled on the membrane, which are characteristic of the blebs of cell necrosis [38], thus, we regarded the blebbing formation as the sign of the cell termination.

In figure 2(b), the minimum energy inducing the blebs was plotted versus the laser spot diameter for the SWCNT-coated dish with various surface resistivity. We found that as the surface resistivity of the SWNTs decreased (the density of SWNTs on the dish increase), the threshold laser power for cell bleb became lower, which clearly indicated that the SWNTs are responsible for a photon antenna, and the higher density of the SWNTs lead to a significant stimuli generation. Interestingly, when the laser spot diameters (50, 25 *μ*m) are close to the cell size (20 ∼ 50 *μ*m) using the 20× and 40× objective lens, the blebs were observed at the lowest laser energy (< 5 *μ*J/pulse), whereas the blebs were observed at the higher energy when the laser spot diameter is much larger (100 *μ*m) or smaller (5, 10 *μ*m) than the cell size. Since the energy densities of the spots were dramatically decreased as the laser diameter increased (figure S4, available at stacks.iop.org/STAM/15/045002/mmedia), it is clear that the threshold values were determined not simply by the energy density. The area of the possible bleb site was also important (figure S5, available at stacks.iop.org/STAM/15/045002/mmedia). Namely, when the spot diameters are much larger (100 *μ*m) than the cell size (20 ∼ 50 *μ*m), in which the energy density is very low, the whole cell areas may produce blebs. In such a case, the threshold values became larger. When the spot diameters were smaller, like 5 ∼ 10 *μ*m, than the cell size, the threshold values are also large due to the small possibility of the bleb generation since the areas of the bleb sites are limited. However, when the laser spot diameters were close, such as 25 ∼ 50 *μ*m, to the cell size, the threshold values were smaller than those of the cases with 100 *μ*m and 5, 10 *μ*m irradiation spot diameters, probably because the whole cell areas become the bleb sites due to the efficient energy density. It is noted that the irradiation of the cell on the glass dish shows no such morphological change even at a laser energy of 250 *μ*J/pulse (figure S6, available at stacks.iop.org/STAM/15/045002/mmedia), which clearly suggests that the SWCNT serves as a photon absorber as well as a stimuli generator to the cell.

**Figure 3.**
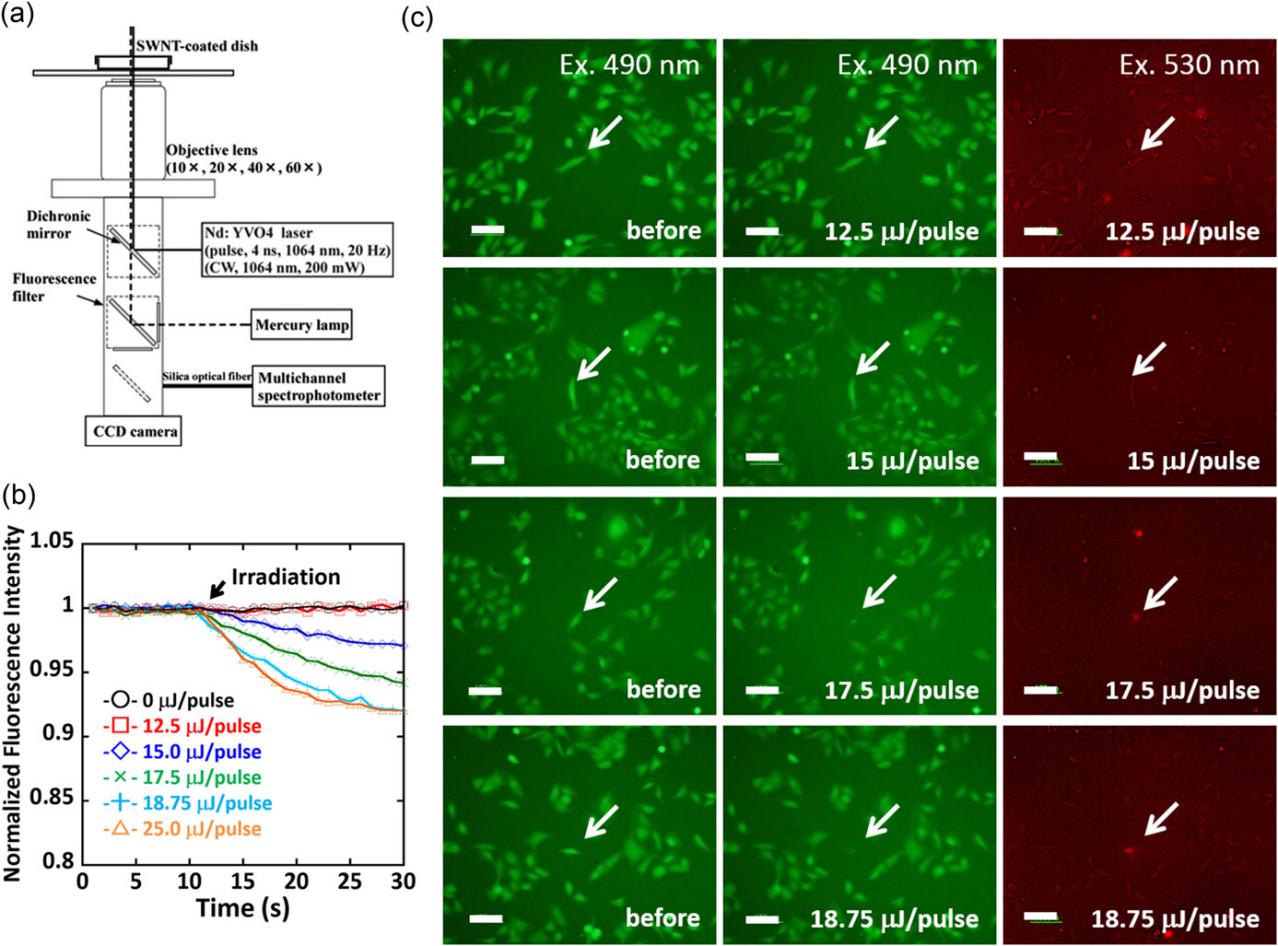
(a) Experimental setup of single cell measurements using a multichannel spectrophotometer. (b) Plots of the normalized fluorescence intensity of calcein-AM in the cells upon irradiation at 0 (black), 12.5 (red), 15.0 (blue), 17.5 (green), 18.8 (light blue) and 25.0 (orange) *μ*J/pulse. (c) Fluorescence microscopy images of the HeLa cells monitored at 490 nm (left and middle column) and 530 nm excitation (right column) before (left column) and after irradiation (middle and right column) at 12.5, 15.0, 17.5 and 18.8 *μ*J/pulse from left to right. Scale bar: 100 *μ*m.

To evaluate the cell viability in detail at around the energy ranges of blebbing formation, the cell was stained using two different dyes, i.e., calcein-AM (*λ*_ex_ = 490 nm, *λ*_em_ = 515 nm) and PI (*λ*_ex_ = 530 nm, *λ*_em_ = 620 nm). Calcein-AM has been widely used to stain living cells and allows us to visualize the change inside living cells via green fluorescence, whereas red fluorescence from PI indicates dead cells [39]. The SWCNT-coated dish with a resistivity of 1 × 10^5^ *Ω* sq^−1^ was selected since no bleb was observed in the widest energy range (0 ∼ 18.8 *μ*J/pulse) among the three substrates. The change in the fluorescence intensity of the single cell of interest irradiated by the NIR pulsed laser was monitored using the setup equipped with a 10× objective lens as depicted in figure 3(a). In this study, six different cells in the media containing both calcein-AM and PI were selected for the irradiation by six different powers (0, 12.5, 15.0, 17.5, 18.8 and 25.0 *μ*J/pulse). Before the irradiation (time = 0 s in figure 3(b)), all the cells were alive and exhibited a strong green fluorescence after staining with calcein-AM (left column in figure 3(c)). Interestingly, the decrease in the fluorescence intensity was observed immediately after the NIR irradiation over 15 *μ*J/pulse (figure 3(b), where the timing of the irradiation is indicated by the arrow). As a matter of fact, the fluorescence images of the irradiated cells (middle column in figure 3(c)) recorded after monitoring the fluorescence intensity (time < 30 s) exhibited decreasing intensity especially for the cells irradiated at 17.5 and 18.8 *μ*J/pulse. On the other hand, red fluorescence from PI staining was observed only from the cells irradiated over 17.5 *μ*J/pulse (right column in figure 3(c); indicated by white arrows). All the above results revealed an unusual behavior of the cell irradiated at 15.0 *μ*J/pulse, where decreasing calcein-AM took place but no staining was observed for PI.

In 2007, Tong *et al* proposed the blebbing mechanism induced by photothermal stimuli as follows; (i) irreversible membrane disruption (perforation) triggered by laser-mediated photothermal stimuli, (ii) influx of extracellular Ca^2+^ through the disrupted membrane and (iii) degradation of the actin cytoskeleton caused by Ca^2+^ [38]. As a result, the blebs were formed when the membrane bilayer was not supported by adhesion to the cytoskeleton [40]. In our study, upon irradiation over 17.5 *μ*J/pulse, the irreversible disruption of the membrane might cause the leakage of calcein-AM together with the entry of the Ca^2+^. A similar threshold nature depending on shear stress (*τ*_max_) produced by the laser-induced cavitation was reported, in which the cells were alive with and without membrane perforation for exposure to shear stress of 8 ± 1 < *τ*_max_ < 18 ± 2 kPa and *τ*_max_ < 8 ± 1 kPa, respectively [41]. The fact agreed well with the threshold nature observed in our fluorescence study, in which the cells were alive with and without membrane perforation for irradiation at 15.0 *μ*J/pulse and <12.5 *μ*J/pulse, respectively, as shown in figure 3(b). Such a perforation without involving cell termination is highly attractive for selective gene transfection, drug injection, regulation of gene expression, etc.

It has been reported that membrane repair is a relatively fast process occurring within a few minutes [42, 43]. Therefore, it is expected that the membrane has repaired before the fluorescence imaging measurements, which were started 10 min after the irradiation.

**Figure 4.**
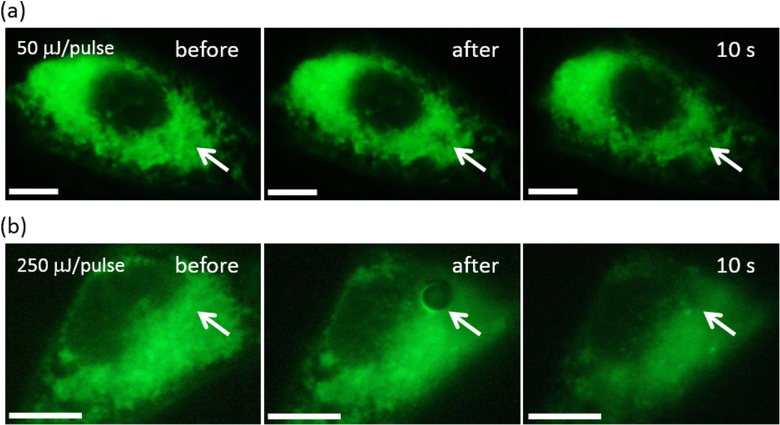
Fluorescence microscopy images of the HeLa cells stained with DiOC_6_ after irradiation at (a) 50 and (b) 250 *μ*J/pulse. Scale bar: 10 *μ*m.

To more clearly study the formation of the irreversible disruption structure in the membrane, the membrane was stained by DiOC_6_, which has often been used to visualize the membranes in the cell [44]. The whole area of the cells was stained green except the area of nucleus as displayed in figures 4(a) (left) and (b) (left) before irradiation. We used a 100× objective lens since the hole was too small for a 10× objective lens to observe. As shown in figure 4(a) (middle), a dark spot due to the perforation, as indicated by a white arrow, was clearly observed in the irradiated area after the 50 *μ*J/pulse irradiation, while at a higher energy (250 *μ*J/pulse) irradiation, a more significant hole was observed (figure 4(b); middle). For these cells, we noticed that the blebbing was induced after the irradiation similar to the cell shown in figure 2(a) (data not shown). Such a successful visualization of the perforation accompanied by the blebbing strongly supported our hypothesis that the fluorescence decrease and the resulting cell termination observed in figure 3(c) (irradiated at 17.5 and 18.8 *μ*J/pulse) was caused by the perforation induced by the pulsed NIR laser irradiation.

Most importantly, the technique presented enabled controlled perforation while keeping the targeted cell alive by tuning the input laser energy as we successfully realized at the 15 *μ*J/pulse irradiation shown in figure 3 using the NIR nanosecond-pulsed laser as a trigger. It is important to note that the energy required for our photo-induced cell manipulation (∼15 *μ*J/pulse) corresponding to about 150 mJ cm^−2^ was much lower than the previous system using gold nanoparticles (about 700 mJ cm^−2^) [17], probably due to the strong photoabsorption of the SWCNTs in the NIR region. Such a system is attractive for various targets, such as selective gene transfection, drug injection, regulation of gene expression, etc, due to high selectivity and softness.

## 4. Conclusions

In conclusion, we demonstrated the perforation of the cell membrane by irradiation with a single low-energy nanosecond pulse from an NIR laser using the SWCNTs as an effective photon absorber as well as a stimuli generator. The cell membrane was reversibly or irreversibly disturbed upon NIR pulse irradiation depending on the energy of the laser. In particular, we observed opening of the cell membrane while keeping the cell alive after the NIR irradiation. Such manipulation of a single cell of interest using a nanosecond pulse allows us to use an inexpensive laser source as a stimulus to engineer the targeted single cell for various targets such as selective gene transfection, drug injection and regulation of gene expression. To demonstrate the significance of our technique, transfection using a plasmid causing expression of green fluorescing protein is under investigation.
